# Selenium and Salt Interactions in Black Gram (*Vigna mungo* L.): Ion Uptake, Antioxidant Defense System, and Photochemistry Efficiency

**DOI:** 10.3390/plants9040467

**Published:** 2020-04-07

**Authors:** Muhammad Jawad Hassan, Muhammad Ali Raza, Imran Khan, Tehseen Ahmad Meraj, Mukhtar Ahmed, Ghulam Abbas Shah, Muhammad Ansar, Samrah Afzal Awan, Nanak Khan, Nasir Iqbal, Yan Peng, Zhou Li

**Affiliations:** 1 Department of Grassland Science, Animal Science and Technology College, Sichuan Agricultural University, Chengdu 611130, China; jawadhassan3146@gmail.com (M.J.H.); Imran.62k@gmail.com (I.K.); Muskanawan62@gmail.com (S.A.A.); pengyanlee@163.com (Y.P.); 2 College of Agronomy, Sichuan Agricultural University, Chengdu 611130, PR China; Razaali0784@yahoo.com (M.A.R.); tehseenahmad55@hotmail.com (T.A.M.); 3 Department of Agronomy, PMAS-Arid Agriculture University, Rawalpindi 46000, Pakistan; ahmadmukhtar@uaar.edu.pk (M.A.); shahga@uaar.edu.pk (G.A.S.); muhammad.ansar@uaar.edu.pk (M.A.); 4 Department of Agricultural Research for Northern Sweden, Swedish University of Agricultural Sciences, Umeå 90183, Sweden; 5 Department of Agronomy, Balochistan Agriculture College, Quetta 87100, Pakistan; Nanakkhan76@gmail.com; 6 School of Agriculture, Food & Wine, The University of Adelaide, PMB1, Glen Osmond, Adelaide 5064, Australia; nasir.iqbal54@gmail.com

**Keywords:** ascorbate-glutathione cycle, micronutrient, fertilization, oxidative damage, chlorophyll, sugar

## Abstract

Salinity is a major abiotic stress which limits crop production, especially under rainfed conditions. Selenium (Se), as an important micronutrient, plays a vital role in mitigating detrimental effects of different abiotic stresses. The objective of this research was to examine the effect of Se fertilization on black gram (*Vigna mungo*) under salt stress. Our results showed that salt stress (100 mM NaCl) in leaves significantly induced oxidative damage and caused a decline in relative water content, chlorophyll (Chl), stomatal conductance (gs), photochemical efficiency (Fv/Fm), sucrose, and reducing sugars. A low dose of Se (1.5 ppm) significantly reduced hydrogen peroxide content, malondialdehyde formation, cell membrane damage, and also improved antioxidative enzyme activities, including superoxide dismutase, catalase, ascorbate peroxidase, glutathione reductase, and glutathione peroxidase under salt stress. Se-treated plants exhibited higher Chl, gs, Fv/Fm, sucrose, and reducing sugars than untreated plants in response to salt stress. In addition, Se application enhanced Se uptake and reduced Na^+^ uptake, but Cl^−^ remained unaffected. Our results indicated that a low dose of Se effectively alleviated salt damage via inhibition of Na^+^ uptake and enhanced antioxidant defense resulting in a significant decrease in oxidative damage, and maintained gaseous exchange and PS II function for sucrose and reducing sugars accumulation in black gram.

## 1. Introduction

Agricultural productivity is adversely affected by soil salinity worldwide. Presently, twenty percent of the world’s arable land and almost half of all irrigated land areas are severely influenced by enormous salt stress. More and more areas are becoming barren because of salt accumulation [1]. The improvement of crop resistance to salinity would increase the food production area of the world tremendously. Adverse effects of salt toxicity on plant physiology result in a reduction of osmotic potential of soil solution and ion toxicity, leading to the disintegration of ionic distribution and water potential in plants [1,2]. A disturbance in the metabolic equilibrium induced by salinity also accelerates oxidative stress resulting in an excessive production of reactive oxygen species (ROS) [3]. The ROS can impart severe damage to numerous biomolecules (e.g., lipid, protein, and DNA) that are crucial for plant existence [4]. Osmotic stress triggered by salinity results in a substantial decrease in stomatal aperture, which reduces photosynthetic capacity [5]. Thus, the antioxidant defense system and photosynthesis are extremely important for plants’ survival under salt toxicity [5,6]. In many plants, reduced growth is closely associated with a decline in photosynthesis under saline conditions [6,7,8]. High salinity inhibits photosynthetic activity of plants associated with stomatal restriction such as stomatal closure [5,9] and non-stomatal restrictions, including denaturation of membrane and enzymatic proteins in photosynthetic apparatus [7,10], chlorophyll (Chl) degradation [8,11], and chloroplast ultrastructure [12]. Plants have a well-organized system of numerous processes involved in the regulation of salt tolerance, such as different types of compatible solutes, antioxidant defense, polyamines, ions uptake, and compartmentalization of injurious ions and ionic transport [13].

Nutrient amelioration has been proposed to mitigate the harmful impacts of salt toxicity in plants [14]. However, these studies have mainly targeted potassium [15,16], calcium [17,18], and phosphorus [16]. Se is an important micronutrient for human beings and livestock [19,20]. A deficiency of Se is responsible for asthma, hypothyroidism, and weakened immune systems in humans [21,22]. The positive effects of Se on plants are concentration or species dependent. Plants are very sensitive to Se (the trace concentration), and higher amounts (>400 µg Sed^−1^) have proven to be drastic and even lethal for plants [23,24]. Due to similar properties, Se and sulphur (S) are absorbed by plants through sulphate transporters and assimilated by the sulfur assimilating pathway [25,26]. Some studies have reported that Se induced beneficial effects on different physiological processes of plants such as improvement in stress tolerance [27] and photosynthesis [28], and alleviation of senescence [29,30]. Previously, if has been shown that Se considerably improved plant growth and physiochemical attributes under salt stress. However, the putative role of Se was dose dependent and varied with its concentration, duration of application, and plant species at different growth stages [31]. The application of Se has enhanced carbohydrate content in the leaves of beans (*Phaseolus vulgaris*) [32] and promoted plant growth and soluble sugar content in coffee (*Coffea arabica*) leaves [33]. Foliar application of Se has increased the quantity and quality of green tea (*Camellia sinensis*) leaves [34]. Se fertilization as selenite to potato tubers has increased protein content and reduced glycoalkaloids, and NO_3_^−^ accumulation [35]. The accumulation of more starch grains and growth improvement have been observed in lettuce plants treated with Se [36]. Se-treated mung bean have also been shown to accumulate more sucrose and starch [37]. For stress tolerance, minute quantities of Se have improved antioxidant enzyme activities and growth during senescence and ultraviolet radiations in lettuce (*Lactuca sativa*) and ryegrass (*Lolium perenne*) [29,38,39]. The application of Se could mitigate the light-induced oxidative damage in potato (*Solanum tuberosum*) [40].

Black gram (*Vigna mungo* L.) belongs to the family of Fabaceae and is one of the most significant economical pulse crops used as a food, green manure, and fodder. Black gram is also a rich and easy source of proteins (20% to 24%), fats (1% to 2%), oil (2.1%), vitamins (A and B), and carbohydrates that are essential for human health [41]. Due to its excellent ability to fix atmospheric nitrogen and convert it to plant useable forms such as nitrates and ammonium ions, black gram plays a vital role in the improvement of soil fertility worldwide [42]. In this study, we focused on the role of different concentrations of Se fertilization in salt-stressed black gram plants on ion uptake, oxidative damage, antioxidative metabolism, photosynthetic functions, and sucrose accumulation. It is beneficial to understand the effects of Se on salt tolerance in crops and investigate farming practices to enhance Se concentration in food commodities.

## 2. Materials and Methods

### 2.1. Planting Material and Treatments

A pot experiment was conducted at the research area of the Agronomy Department PMAS Arid Agriculture University, Rawalpindi from March to June 2018. The research area was located at 33.6492° N latitude and 73.0815° E longitude. The experiment was laid out in plastic pots with 18 cm diameter and 22 cm depth. Each pot contained 5 kg loam, 10 milligram Ca_3_(PO_4_)_2_ kg^−1^, and 0.5 Kg farmyard manure, respectively. All pots were arranged under a completely randomized design (CRD). After filling, each pot was treated with a salt solution (100 mM NaCl) until its saturation and dried out for two days. Later, the sodium selenate (Na_2_SeO_4_) was dissolved in distilled water (1.5, 3, and 4.5 ppm) and irrigated in fully dried soil before sowing. Seeds of black gram cultivar “Chakwal Mash” were obtained from the National Agriculture Research Centre and used as plant material. Before sowing, seeds were washed with 75% ethanol and deionized water for 5 min. Ten seeds were sown in each pot, and after thinning, three plants were maintained in each pot. Plants were well irrigated daily to prevent a risk of water stress in a greenhouse (average temperatures of 25/20 °C (day/night) and 800 μmoL m^−2^ s^−1^ photosynthetically active radiation). At the flowering stage (35 days after sowing), the fully expanded and mature leaves from each treatment were selected for assessment of physiological parameters. Each treatment was thrice replicated (non-saline control, salt stress, salt + 1.5 ppm Se, salt + 3 ppm Se, and salt + 4.5 ppm Se), and a total of 15 pots (each pot contained three plants) was used for the above treatments.

### 2.2. Determination of Se, Na, and Cl Concentrations

Leaf samples were washed with deionized water, blotted to eliminate extra water, and then dried in an oven. Dried samples were thoroughly homogenized with mortar and pestle, shifted to a digestion flask consisting of ten milliliters of 4 M HNO_3_, and placed at room temperature overnight. After being heated at 125 °C for 4 hrs, solutions were diluted (50 mL) and cooled for further measurements. The Se concentration (µg g^−1^ DW) was measured in triplicate using atomic absorption spectrometry [43]. For Na and Cl determination, samples were dried and ground to powder form, and then shifted to an Erlenmeyer flask containing 6 mL of HClO_4_ and HNO_3_ solution. The flasks were placed for 30 min at 40 °C in a water bath until condensed to 1 milliliter extract by heating at 150 to 180 °C. Distilled water was used to dissolve this residue to the final volume of 100 mL. The Na concentration in the leaf samples was estimated by using flame photometry, whereas Cl^-^ was determined by precipitation titration with Ag_2_NO_3_ following Mohr’s method [44].

### 2.3. Determination of Leaf Water Status

Leaf samples were detached to calculate the relative water content (RWC) following the procedure described by [45]. Fresh weight (FW) was noted instantly and dipped in deionized water overnight to obtain the turgid weight (TW). Samples were placed in an electric oven for one day at 75 °C to obtain dry weight (DW). The RWC was estimated using the following equation: RWC (%) = 100 × [(FW – DW)/(TW – DW)].

### 2.4. Measurement of Chlorophyll Content, Stomatal Conductance, and Photochemical Efficiency

In order to examine the Chl content, 1 g fresh leaf samples were ground in 80 percent CH_3_COCH_3_, and centrifuged at 1816*g* for 10 min. The supernatant was obtained, and an absorbance value at 645 and 663 nm was noted to measure the total Chl content [46] against 80 percent acetone as a blank. Stomatal conductance (gs) was estimated from fully expanded, mature, and healthy leaves using a portable leaf porometer (model SC1, Decagon Devices, Pullman, WA, USA) at 11.00 a.m. and given as mmol m^−2^ s^−1^.

Leaves were obtained from the top second or third branch, and photochemical efficiency was estimated as Chl fluorescence following a dark-adapted test of modulated Chl fluorometer (OS1-FL, Opti-Sciences, Tyngsboro, MA, USA) at 11:00 h. The fluorometer provided a solid-state light source of 660 nm and had filters to block radiations above 690 nm. The mean intensity of this modulated light was set from 0 to 1 mE. A PIN silicon photodiode was used for detection in the range 700–750 nm with adequate filtering to eliminate extraneous light. Leaves were placed in the instrument clamps under darkness to cease photosynthesis (light reaction) for 45 min. Clamps were connected with the optic fiber of the device, and clamps valves were opened. After the onset of the device, the modulated light of 695 nm was radiated towards the leaf through the optic fiber. Photosystem II (PSII) activity was given as the Fv/Fm ratio.

### 2.5. Determination of Oxidative Damage and Cell Membrane Stability

Lipid peroxidation was estimated as the malondialdehyde (MDA) content according to the procedure of [47]. Then, 0.5 g of leaf tissue samples were homogenized in 5 milliliters of 5% TCA. Homogenate was centrifuged for 10 min at 25 °C, heated at a high temperature (98 °C) for 10 min, and allowed to cool in ice immediately. The absorbance value was estimated at a wavelength of 532 nm. For the estimation of hydrogen peroxide (H_2_O_2_) concentration, protocols involving potassium iodide (KI) were used. Absorbance values were noticed at 390 nm. The quantity of hydrogen peroxide produced was obtained by following the standard curve drawn with previously known readings of hydrogen peroxide [48]. In order to measure cell membrane stability, leaf samples (0.2 g) were collected, rinsed with distilled water to eliminate attached electrolytes, and kept in vials that contained ten milliliters of distilled water. Closed vials were placed at 25 °C for six h, and electrical conductivity (C1) of the solution was determined. After this, samples were kept in an electric oven at 90 °C for two h, and the electrical conductivity (C2) of solution was estimated. Electrolyte leakage (EL) was measured in percent as follows: EL% = 100 × EC1/EC2.

### 2.6. Determination of Antioxidant Enzyme Activities

Leaf tissue was frozen and subsequently homogenized in 4 mL of a solution having 1% (*w/v*) polyvinylpolypyrrolidone (PVP), 50 mM phosphate buffer (pH 7.0), and 0.2 mM ascorbic acid. Then, the homogenate was centrifuged for 30 min at 15,000*g*. The supernatant was rapidly desalted at 4 ºC by passing it through four milliliters Sephadex G-25 columns pre-equilibrated with a buffer consisting of 0.05% BSA, 0.25 mM MgCl_2_, 1 mM EDTA, 20 mM HEPES-NaOH (pH 7.5), and 0.01% 2-mercaptoethanol. All events were completed at 0 to 4 °C. The SOD activity was measured according to the method of [49]. The reaction mixture comprised of 13 mM methionine, 2 mM riboflavin, 5 mL enzyme extract, 50 mM phosphate buffer (pH 7.8), 75 mM *p*-nitroblue tetrazolium chloride (NBT), and 0.1 mM EDTA. One unit of SOD activity was defined as the concentration of the SOD enzyme needed for 50% inhibition of nitro blue tetrazolium reduction. The CAT activity was measured as a decline in absorbance value at 240 nm for 1 min resulting in the disintegration of H_2_O_2_ following the procedure of [50]. The assay medium consisted of 15 mM H_2_O_2_ and 50 mM phosphate buffer (pH 7.0).

The APX activity was estimated as a reduction in absorbance value at 290 nm for 1 min according to the procedure of [51]. The estimation mixture included 0.1 mM H_2_O_2_, 50 mM sodium phosphate buffer (pH 7.0), 0.5 mM ASA, 0.1 mM EDTA, and 0.15 mL enzyme extract. The GR activity was examined, as explained by [52]. Then, oxidized GSH (GSSG)-dependent NADPH oxidation was followed at 340 nm in 1 mL of reaction mixture including 0.1 mM NADPH, 100 mM sodium phosphate buffer (pH 7.8), 50 µL extract, and 0.5 mM GSSG. The GPX activity was determined using the protocol of [53] with H_2_O_2_ as a substrate. The reaction mixture included 1 mM NaN_3_, 1 mM EDTA, 1 U glutathione reductase, 25 µL sample solution, 100 mM Na-phosphate buffer (pH 7.5), 0.12 mM NADPH, 2 mM GSH, and 0.6 mM H_2_O_2_. The reaction was initiated by adding H_2_O_2_. The NADPH oxidation was measured at 340 nm for 1 min.

### 2.7. Determination of Sucrose and Reducing Sugars

The sucrose content was estimated by following the procedure described by [54]. Leaf tissue was extracted using 80 percent ethanol and repeated three times at 80 °C for 1.5 hours for each extraction. The extract was collected and vaporized in an oven at 40 °C. Then, 200 mL of aliquots from samples and standard sucrose were mixed with the reaction solution (1 mL) which consisted of 0.4 mM NADP+, 5 mM MgCl_2_, 0.02% (weight/volume) BSA, 2 mg/mL yeast hexokinase (EC 2.7.1.1), 100 mM imidazole buffer (pH 6.9, 60 mM imidazole HCl, 40 mM imidazole base), 1 mM ATP, 0.5 mM dithiothreitol, 20 mg/mL yeast invertase (EC3.2.1.26), and 1 mg/mL yeast phosphoglucoisomerase (EC 5.3.1.9) and incubated at 25 °C for 30 min to allow conversion of fructose and glucose to glucose-6-phosphate, and readings were noted at 340 nm. After 85 milliliters of C_6_H_12_O_6_-6-PO_4_^−2^ dehydrogenase (70 units/mL) was added, the mixture was blended thoroughly. Absorbance was again recorded when the readings became stable after 5 min. Blanks were run using 1 milliliter of the reaction mixture and 200 milliliters of the extract without invertase. A standard curve was used to convert values obtained from the samples to sucrose concentrations and given as µmol/g (DW). To assess the reducing sugars, the DNSA reagent (1 mL 3,5-dinitrosalicylic acid) was mixed with ethanol extract (1 mL). The reaction mixture was heated for 12 min, and then 2 mL of distilled water was added. The absorbance was noted against a blank consisting of 80% C_2_H_5_OH instead of C_2_H_5_OH extract. The content of the reducing sugars was estimated using a standard curve.

### 2.8. Statistical Analysis

Statistical analysis was conducted using statistix 8.1 (version, 8.1. Statistix, USA). Significant differences among all treatments were measured by using ANOVA (one way) in combination with LSD (least significant difference) test. The significance of differences was evaluated at the 5 percent probability level (*p* < 0.05).

## 3. Results

### 3.1. Effects of Exogenous Se on Se, Na, and Cl Uptake under Salt Stress

**Figure 1:**
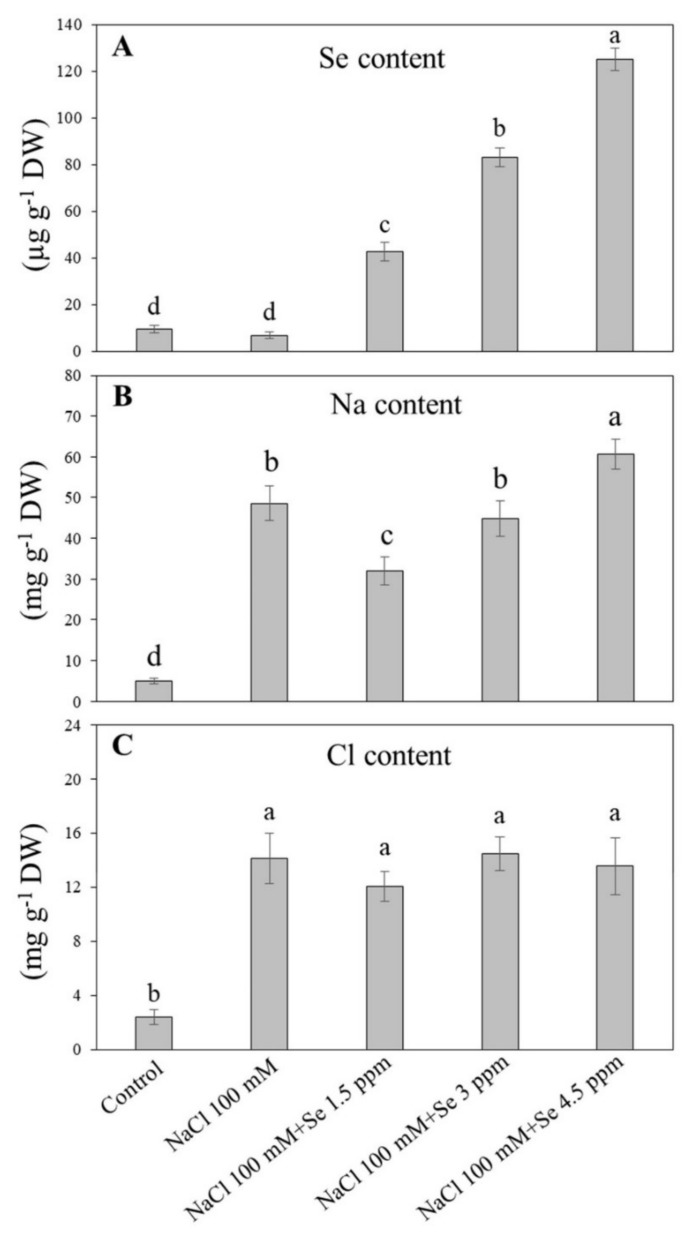
Effect of selenium (Se) fertilization on (**A**) Se uptake; (**B**) sodium (Na) uptake; and (**C**) chloride (Cl) uptake in leaves if black gram plants under salt stress. Values are mean ± standard error (*n* = 3). Different letters in vertical columns show a significant difference. A comparison of mean was confirmed by LSD at *p* < 0.05.

Salt stress did not affect Se accumulation, but exogenous Se fertilization significantly enhanced Se uptake in leaves of black gram (Figure 1A). For all exogenous Se treatments, the highest Se accumulation (125.133 µg g^−1^ DW) was observed in treatment (NaCl 100 mM + Se 4.5 ppm), second (83.233 µg g^−1^ DW) in treatment (NaCl 100 mM + Se 3 ppm), and lowest (42.667 µg g^−1^ DW) in treatment (NaCl 100 mM + Se 1.5 ppm), which showed a progressive trend in Se accumulation with an increase in its applied concentrations (Figure 1A). The application of NaCl significantly increased Na uptake in leaves (Figure 1B). In combined applications of Se and NaCl, only a lower concentration of Se (1.5 ppm) significantly restricted Na accumulation in leaves; the three ppm of Se had no significant effect on Na uptake. In comparison, the higher level (4.5 ppm) significantly increased Na accumulation in leaves of black gram. As compared to the control, salt stress and salt stress combined with exogenous Se (1.5, 3, and 4.5 ppm) application induced significant increases in Cl accumulation, but no significance in Cl uptake were observed among these four treatments (NaCl, NaCl + 1.5 ppm Se, NaCl +3 ppm Se, and NaCl+4.5 ppm Se) (Figure 1C).

### 3.2. Effects of Exogenous Se on Oxidative Damage and Cell Membrane Stability under Salt Stress

**Figure 2:**
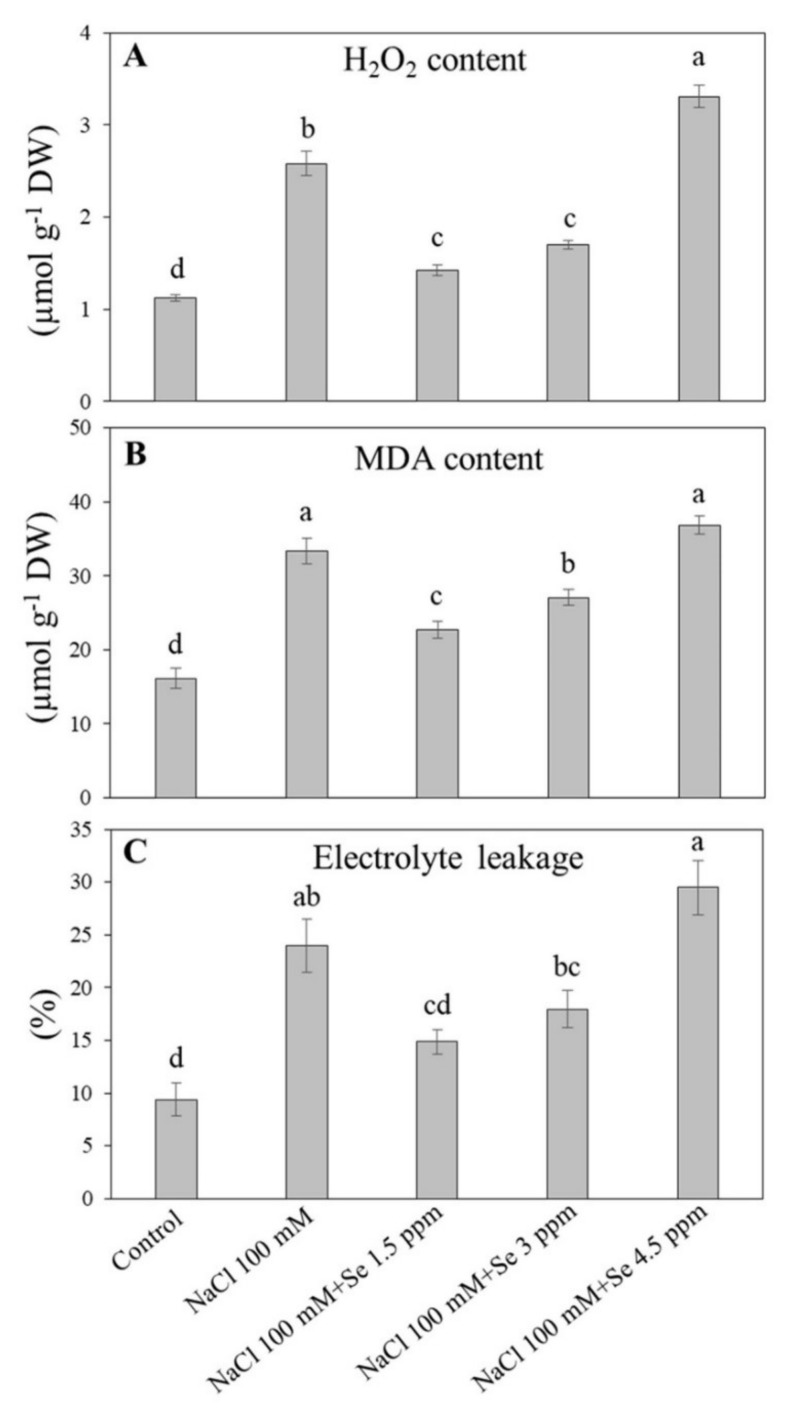
Effect of selenium (Se) fertilization on (**A**) hydrogen peroxide (H_2_O_2_); (**B**) malondialdehyde (MDA) content; and (**C**) electrolyte leakage (EL) in leaves of black gram plants under salt stress. Values are mean ± standard error (*n* = 3). Different letters in vertical columns show a significant difference. A comparison of mean was confirmed by LSD at *p* < 0.05.

Salt stress (NaCl 100 mM) significantly influenced H_2_O_2_ concentration in leaves of black gram plants. However, low and intermediate Se application significantly reduced H_2_O_2_ with respect to Se-free saline treatment, whereas it was even increased at the high dose (Figure 2A). Lipid peroxidation, as reflected by MDA content, was greatly enhanced by salt stress in black gram plants (Figure 2B). Among all treatments, higher MDA content was noticed in the treatment (NaCl 100 mM + Se 4.5 ppm) and salt stress, while the lowest (16.100 µmol g^−1^ DW) in the control, which suggested that Se at high concentrations was toxic for black gram plants under saline conditions. Salt treatment (NaCl 100 mM) significantly elevated MDA content in black gram plants as compared with the control. The MDA content was significantly reduced by Se treatment (NaCl 100 mM + Se 1.5 or 3 ppm) as compared with salt stress (NaCl 100 mM) (Figure 2B). Salt stress significantly increased the level of EL up to (24%) in contrast to the control. The low concentration of Se (NaCl 100 mM + Se 1.5 ppm) reduced EL in relative to salt treatment (NaCl 100 mM). EL exhibited an increasing trend from lower (1.5 ppm) to higher doses of Se (4.5 ppm) application under salt stress (Figure 2C).

### 3.3. Effects of Exogenous Se on Antioxidant Defense under Salt Stress

**Figure 3:**
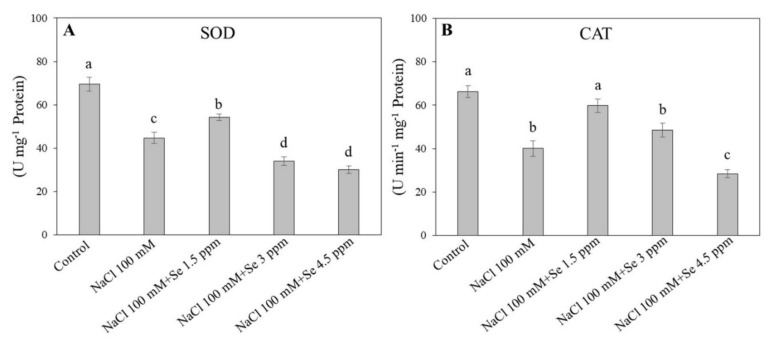
Effect of selenium (Se) fertilization on (**A**) superoxide dismutase (SOD) and (**B**) catalase (CAT) activities in leaves of black gram plants under salt stress. Values are mean ± standard error (*n* = 3). Different letters in vertical columns show a significant difference between each treatment. A comparison of mean was confirmed by LSD at *p* < 0.05.

**Figure 4:**
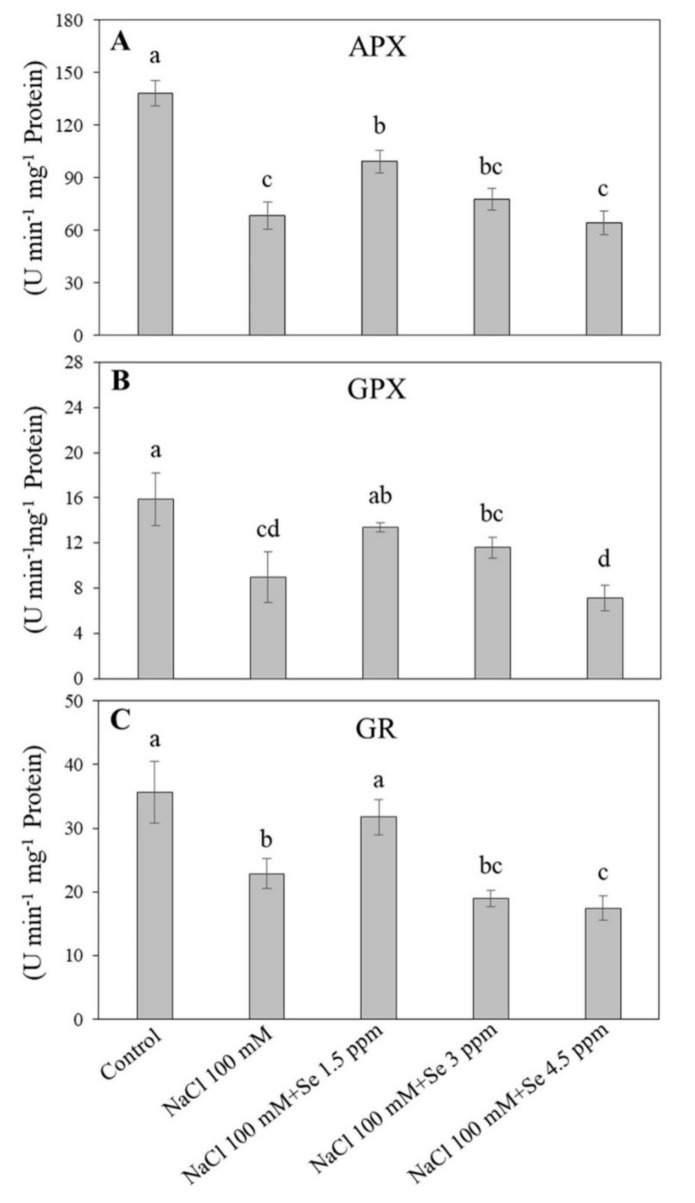
Effect of selenium (Se) fertilization on (**A**) ascorbate peroxidase (APX); (**B**) glutathione peroxidase (GPX); and (**C**) glutathione reductase (GR) in leaves of black gram plants under salt stress. Values are mean ± standard error (*n* = 3). Different letters in vertical columns show a significant difference. A comparison of mean was confirmed by LSD at *p* < 0.05.

Salt stress negatively influenced the defensive system of black gram plants by impairing the antioxidant enzyme activities (Figure 3 and Figure 4). The SOD and CAT activities in the control were found to be maximum, whereas salt stress reduced their activities (Figure 3A). An improvement in SOD and CAT activities was observed in the treatment (NaCl 100 mM + Se 1.5 ppm) as compared with the salt treatment (NaCl 100 mM) in leaves. The higher dose of Se (3 and 4.5 ppm) reduced SOD and CAT activities along with the salt application (Figure 3). Similar trends were noticed in changes of APX, GPX, and GR activities (Figure 4). The Se (1.5 ppm) application improved the APX activity in leaves by 30.89% under salt stress as compared with the salt treatment (NaCl 100 mM). Significant declines in GPX and GR activities were found under the 100 mM NaCl treatment as compared with the control (Figure 4B,C). The maximum GPX (15.867 U min^−1^ mg^−1^ protein) and GR (35.600 U min^−1^ mg^−1^ protein) activities were recorded in the control, while the minimum GPX ( 8.967 µ guaiacol min^−1^ mg^−1^ protein) and GR (22.867 µmoL NADPH min^−1^ mg^−1^ protein) activities were measured in the treatment (NaCl 100 mM + Se 4.5 ppm), respectively. The addition of Se at the 1.5 ppm dose maintained GPX and GR activities at normal levels (Figure 4B,C).

### 3.4. Effects of Exogenous Se on Leaf Water Status and Photochemical Efficiency under Salt Stress

**Figure 5:**
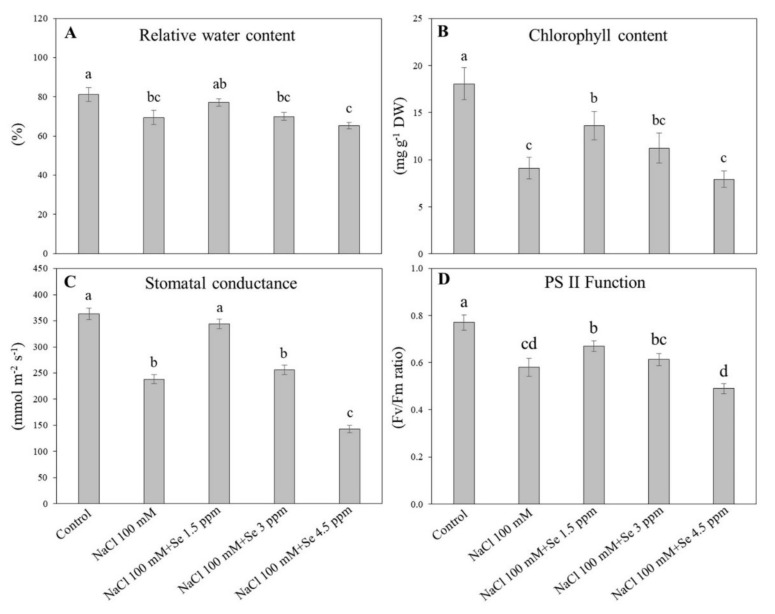
Effect of selenium (Se) fertilization on (**A**) relative water content (RWC); (**B**) chlorophyll (Chl); (**C**) stomatal conductance (gs); and (**D**) photochemical efficiency (Fv/Fm) in leaves of black gram plants under salt stress. Values are mean ± standard error (*n* = 3). Different letters in vertical columns show a significant difference. A comparison of mean was confirmed by LSD at *p* < 0.05.

The highest RWC (81.167%) was noted in the control and the lowest RWC (65.333%) was detected in the treatment (NaCl 100mM + Se 4.5 ppm) (Figure 5A). The RWC in the salt treatment (NaCl 100 mM) was significantly decreased as compared with the control. The Se at a lower dose (1.5 ppm) with salt application significantly improved the leaf RWC as compared with the salt only treatment (100 mM); however, the higher doses of Se with salt decreased the leaf RWC drastically (Figure 5A). Different applications of Se and NaCl significantly affected total Chl content in black gram leaves (Figure 5B). Salt stress significantly decreased the total Chl content, but Se application at a lower level (1.5 ppm) alleviated the salt-induced decline in total Chl content in black gram plant leaves (Figure 5B). In contrast, a significant reduction in total Chl content was noticed when the Se concentration was increased beyond 1.5 ppm under salt stress (Figure 5B). NaCl (100 mM) treatment significantly reduced gs by 34.35% as compared with the control. The treatment (NaCl 100 mM + Se 1.5 ppm) showed a 30.76% increase in gs as compared with the salt treatment (NaCl 100 mM) (Figure 5C). The higher dose of Se (4.5 ppm) under salt stress significantly inhibited gs as compared with the salt treatment (Figure 5C). The maximum Fv/Fm (0.770) for photosystem II was observed in the control, whereas the minimum Fv/Fm (0.490) was detected in the treatment (NaCl 100 mM + Se 4.5 ppm). The salt only treatment (NaCl 100 mM) significantly reduced Fv/Fm by 24.67% as compared with the control. A low dose of Se (1.5 ppm) significantly alleviated the harmful effects of salinity in leaves of black gram plants (Figure 5D).

### 3.5. Effects of Exogenous Se on Sugar Accumulation under Salt Stress

**Figure 6:**
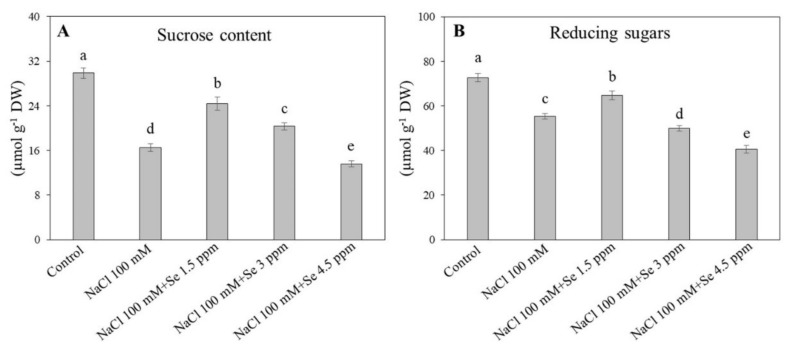
Effect of selenium (Se) fertilization on (**A**) sucrose content and (**B**) reducing sugars in leaves of black gram plants under salt stress. Values are mean ± standard error (*n* = 3). Different letters in vertical columns show a significant difference. A comparison of mean was confirmed by LSD at *p* < 0.05.

**Figure 7:**
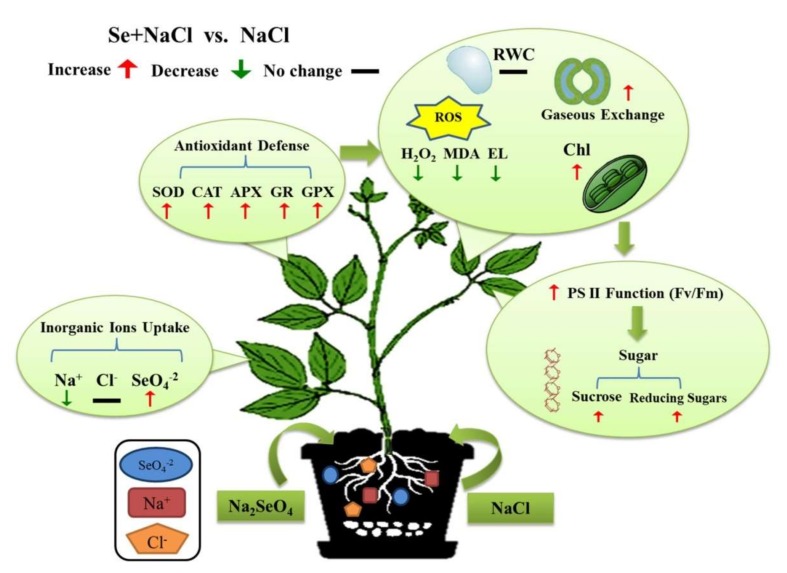
Comprehensive schematic diagram presenting the protective role of selenium (Se) at an optimum concentration (1.5 ppm) in leaves of black gram under salt stress.

Salt stress and Se application significantly affected sucrose concentration in leaves of black gram plants (Figure 6A). In contrast to the control plants, sucrose concentration in leaves significantly decreased by 54.57% in plants growing in salt stress (NaCl 100 mM) (Figure 6A). The combined treatment (NaCl 100 mM + Se 1.5 ppm) significantly alleviated the harmful impact of salinity and successfully improved sucrose concentration by 32.28% as compared with the salt treatment (NaCl 100 mM). Moreover, the treatment (NaCl 100 mM + Se 3 ppm) maintained significantly higher sucrose content than the salt treatment. The treatment (NaCl 100 mM + Se 4.5 ppm) significantly reduced sucrose content as compared with the salt only treatment (Figure 6A). Reducing sugars significantly declined in leaves under salt stress in spite of the Se application (Figure 6B). In contrast to the control, the salt treatment significantly decreased the reducing sugar level by 23.78%, but the addition of Se (NaCl 100 mM + Se 1.5 ppm) significantly mitigated the adverse effects of salt stress on reducing sugars. The concentration of Se beyond 1.5 ppm (3 and 4.5 ppm) exhibited toxic effects and adversely influenced reducing sugars accumulation in the leaves of black gram plants under toxic salt conditions (Figure 6B). Figure 7 presented the protective role of Se at an optimum concentration (1.5 ppm) in leaves of black gram under salt stress.

## 4. Discussion

Salinity is extremely harmful to crops belonging to the family of Fabaceae such as mungbean (*Vigna radiata*), soybean (*Glycine max*), and many other pulse crops, resulting in reduced growth and yield [5]. On the basis of previous studies about the advantageous roles of Se on plants under stressed conditions [55], we examined the role of Se application in black gram plants under salt-stressed soil. Interestingly, the results showed that only 1.5 ppm of Se supplements under salt-stressed soil improved the performance of various physiological and biochemical parameters in black gram plants, whereas 3 and 4.5 ppm dose amplified damage under salt stress. In addition, the plants uptake of Se was not affected due to salt stress indicating that Se was probably not a salt-responsive element in black gram. As expected, salt stress significantly increased Na and Cl ions in leaves of black gram with more Na than Cl ions. Similar findings were reported in some grass species [56] and *Avicennia germinans* [57]. To date, it is unknown which element (Na or Cl) is more responsible for NaCl-induced ionic stress in black gram. It has been reported that Se application at low doses (5 and 10 μM) decreased Cl content in cucumber plants under salt stress [58]. Exogenous Se significantly reduced Na accumulation in roots when maize (*Zea mays*) suffered from salt stress [59]. To date, limited information is available about Na and Se antagonism in legumes grown under salt-stressed soil. In the present study, the application of Se (1.5 ppm) significantly increased Se accumulation in leavs and decreased Na uptake with no significant effect on Cl uptake under salt stress. These results indicated that an appropriate dose of Se (1.5 ppm) effectively alleviated salt damage due to the inhibition of Na uptake in black gram.

Under salt stress, plants accumulate high levels of reactive oxygen species that are extremely dangerous for various cellular components, including lipids, nucleic acids, proteins, and pigments leading to membrane lipid peroxidation and decline in membrane stability [60,61]. Membrane integrity and stability could be estimated by MDA content and EL level [62]. Salt-tolerant wheat (*Triticum aestivum*) and tomato (*Solanum lycopersicum*) demonstrated lower H_2_O_2_ and MDA content in contrast to susceptible ones [63,64]. In addition, Se application could reduce the MDA content in soybean during senescence [30]. Our study demonstrated that salt stress significantly increased H_2_O_2_ and MDA content and EL level. However, membrane damage in black gram plants affected by salt stress was observed to be significantly decreased in leaves fertilized with 1.5 ppm dose of Se, which is in accordance with previous reports [14,58]. It has been reported that Se application improved antioxidant enzyme activities, including SOD, POD, and GR, and alleviated oxidative damage in various plants under saline conditions [6,14,59]. In our study, significant promotion in activities of SOD, CAT, APX, GR, and GPX were detected in salt-Se-treated black gram plants (NaCl 100 mM + Se 1.5 ppm) in contrast to salt-affected plants (NaCl 100 mM). These findings were in agreement with previous results where Se improved antioxidant defense in sorrel seedlings under salt stress [14]. Different antioxidants such as SOD, CAT, APX, GPX, and GR are extremely important for ROS scavenging in plants under salt-stressed conditions. SOD is the integral and front line in antioxidant defense against oxidative damage by converting superoxide radical (O_2_^−^) into hydrogen peroxide and molecular oxygen [65]. CAT is principally involved in the removal of surplus H_2_O_2_ [55,66]. When Se is present, GPX and GR are more efficient in eliminating H_2_O_2_ as compared with APX and CAT [6]. GPX, APX, and GR are crucial globular proteins of the ascorbate-glutathione cycle playing important roles in tandem with ascorbate peroxidase to scavenge H_2_O_2_ [66], which have been described to be stimulated markedly in plants growing under stressed abiotic conditions [43,67,68]. In the present study, a low dose of Se (1.5 ppm) improved salt tolerance in black gram through enhancement of the antioxidant defense, thereby alleviating the salt-induced membrane lipid peroxidation and enhancing membrane stability. In addition to the advantageous roles of Se application at a lower concentration (1.5 ppm), higher levels (3 and 4.5 ppm) exacerbated harmful effects caused by salinity, which could be associated with the considerable accumulation of Se and Na ions in leaves. The present reports are in accordance with earlier findings with respect to the interactive roles of Se and arsenic and higher concentrations of Se assisted arsenic uptake [69].

Salt stress induces physiological drought resulting in a decline in leaf water potential, RWC, stomatal conductance, and ultimately transpiration. The current study found that black gram plants exposed to salt treatment exhibited a significant reduction in RWC, which probably was related to a hindrance in water uptake. Salt induced reduction in RWC has been observed in triticale [70], safflower [71], and sorghum [72]. An inhibition in gs was also observed in this study, which is similar to results reported in salt-affected mustard (*Brassica* spp.) [73] and barley (*Hordeum vulgare*) [74]. Interestingly, the application of Se alleviated declines in the gs of black gram leaves caused by salt. Similar results of Se on RWC and gs have been found in potato and olive (*Olea europaea*) under drought stress [75,76]. Water status and gaseous exchange through stoma are directly related to photosynthesis. A decline in Chl content and fluorescence causes a significant reduction in photosynthetic function when plants respond to salt stress. The photosynthetic rate has been significantly improved by Se application in sorghum under heat stress [77]. Se application has been associated with amelioration in photosynthesis and Chl protection and has been observed in sorrel (Rumex patientia × R. tianshanicus) [14] and cucumber [58] under saline conditions. Our study showed that salt stress severely affected the photosynthetic process by causing a significant reduction in Chl content and Fv/Fm in non-Se-treated black gram plants, whereas Se-treated (1.5 ppm) black gram plants exhibited higher Chl content and Fv/Fm under salt stress. Se application has been reported to protect the function of Chl and photosystem II from salt-induced oxidative damage through improvement in antioxidant enzyme activities in tomato seedlings [6], which supports our findings. Under salt stress, a disturbance in sucrose metabolism, as demonstrated by a decrease in sucrose and reducing sugar content in leaves of salt-exposed black gram plants, could inhibit photosynthesis in leaves. Salt stress has been described to have a negative impact on sucrose metabolism by influencing genes and enzymes associated with sucrose synthesis in tomato [78]. However, we observed that the Se application significantly restricted salt-induced decline in sucrose and reducing sugars in black gram leaves. An earlier study found that 0.75 ppm of Se improved sucrose and reducing sugar content, and thus provided more energy for mungbean (*Phaseolus aureus.*) growth under normal conditions [37]. There is a need to further explore the contribution of Se-regulated sugar metabolism to salt tolerance in black gram.

## 5. Conclusions

The current study exposed that the addition of Se at a low concentration (1.5 ppm) to black gram plants growing in saline soil hindered Na uptake, resulting in amelioration of physiological and biochemical responses such as antioxidant defense, Chl concentration, gs, photochemical efficiency, sucrose levels, and reducing sugars. Se improved antioxidant enzyme activities (SOD, CAT, APX, GPX, and GR), followed by a significant reduction in ROS accumulation and membrane lipid peroxidation in salt-stressed black gram plants, which could be beneficial to the maintenance of higher Fv/Fm and sugar content under salt stress. Se fertilization at a low concentration (1.5 ppm) could be one of the potential approaches to minimize the effects of salt toxicity in black gram plants growing under salt-affected soils.
